# Structured multi-level knowledge augmentation via small-to-large evidence-guided collaboration for knowledge-based VQA

**DOI:** 10.3389/frai.2026.1884321

**Published:** 2026-07-27

**Authors:** Meng Zhang, Dayu Wu, Wonjun Chung

**Affiliations:** 1 College of Architecture and Design, Tongmyong University, Busan, Republic of Korea; 2 Department of Visual Communication Design, Yeungnam University, Gyeongsan, Republic of Korea

**Keywords:** entity enhancement, knowledge-based visual question answering, large–small model collaboration, LLM, multimodal reasoning

## Abstract

Knowledge-based visual question answering requires models to connect question-relevant visual evidence with external knowledge for accurate reasoning. However, existing approaches still face two critical challenges: insufficient alignment between visual content and question intent, which leads to missing or irrelevant evidence, and ambiguity in entity-level semantics, especially for fine-grained or knowledge-intensive concepts. To address these issues, we propose an inference-time evidence augmentation framework for frozen-LLM-based KB-VQA. The framework uses lightweight vision–language models to construct structured textual evidence, which is subsequently provided to a frozen large language model (LLM) for final reasoning. We emphasize that the proposed method does not introduce a new LLM architecture or a new training mechanism; instead, it focuses on how question-relevant multimodal evidence can be systematically constructed, refined, and organized before LLM inference. The framework consists of four complementary evidence-construction modules: (1) a question-oriented image information extraction module that generates query-relevant visual descriptions to enhance visual–semantic alignment; (2) an entity enhancement module that introduces clarifying sub-questions to alleviate entity-level ambiguity; (3) a candidate-guided answer generation module that provides plausible answer cues to constrain the reasoning space; and (4) a contextual exemplar retrieval module that supplies relevant demonstrations to support knowledge-grounded inference. Experiments on OK-VQA and A-OKVQA show that the proposed method achieves 66.72% and 69.51% accuracy, respectively, outperforming strong baselines, while supplementary analyses examine its robustness, output-format reliability, and inference cost.

## 1 Introduction

With the rapid advancement of artificial intelligence, the integration of vision and language has become a central topic in multimodal learning. Among various multimodal tasks, Visual Question Answering (VQA) serves as a representative benchmark that requires models to answer natural language questions grounded in visual inputs (Antol et al., 2015; Goyal et al., 2017; Krishna et al., 2017; Hirota et al., 2024). In its knowledge-based setting, the task further requires models to connect fine-grained visual evidence with external knowledge and semantic reasoning (Marino et al., 2019, 2021). VQA has been widely adopted as a testbed for measuring multimodal intelligence.

The development of VQA has undergone several stages. Early studies mainly relied on task-specific multimodal fusion and attention architectures (Kim et al., 2018; Ben-younes et al., 2017). Knowledge-intensive extensions then incorporated explicit knowledge bases or symbolic reasoning (Wang et al., 2018; Marino et al., 2021). However, these methods often remained limited in multi-step reasoning, compositional understanding, and open-domain knowledge-intensive scenarios. With the emergence of large-scale vision–language pretraining, VQA gradually shifted from task-specific design to general-purpose multimodal representation learning. Models such as ViLBERT and LXMERT (Lu et al., 2019; Tan and Bansal, 2019) learned stronger cross-modal representations, while VinVL and BLIP (Zhang et al., 2021; Li et al., 2022) further improved visual encoders and pretraining strategies. More recent multimodal foundation models, including BLIP-2, PaLI, and Flamingo (Li et al., 2023; Chen et al., 2023; Alayrac et al., 2022), as well as instruction-tuned systems such as InstructBLIP, LLaVA, and MiniGPT-4 (Dai et al., 2023; Liu et al., 2023; Zhu et al., 2023), have substantially enhanced few-shot and zero-shot multimodal reasoning. In parallel, large language models (LLMs) have introduced a new paradigm for VQA by offering strong language understanding and in-context learning capabilities (Brown et al., 2020; de Zarzà et al., 2026). Existing LLM-based VQA approaches can be broadly grouped into three lines. Prompting-based methods guide answer generation through designed prompts or answer heuristics (Yang et al., 2022; Tiong et al., 2022; Shao et al., 2023). Retrieval-augmented or knowledge-validated methods improve reasoning by introducing external examples, contextual knowledge, or structured validation signals (Ravi et al., 2023; Wu et al., 2022; Hu et al., 2024). Hybrid multimodal frameworks combine visual perception with LLM-based reasoning to enable more flexible cross-modal inference (Tsimpoukelli et al., 2021; Alayrac et al., 2022; Liu et al., 2023). Despite their effectiveness, these approaches are still sensitive to prompt quality and contextual inputs, and many of them underutilize fine-grained multimodal evidence during reasoning (Wang et al., 2023). Although recent advances have significantly improved VQA, a critical research gap remains: *existing approaches still lack a unified mechanism that effectively integrates fine-grained visual perception, entity-level semantics, and structured contextual reasoning*. Many methods either rely mainly on pretrained global representations or depend heavily on prompting strategies, without explicitly coordinating perception and reasoning within a single framework. More specifically, three key limitations can be identified:

- *Insufficient visual–semantic alignment*: even advanced multimodal encoders such as CLIP (Radford et al., 2021; Onuoha et al., 2024) often produce coarse or generic representations that are weakly aligned with the intent of a given question. Consequently, task-relevant visual evidence may be overlooked, leading to suboptimal reasoning performance.
- *Ambiguity in entity semantics*: knowledge-based VQA frequently involves fine-grained, domain-specific, or culturally dependent entities. Existing models struggle to distinguish semantically similar concepts, and external knowledge resources such as ConceptNet, Wikidata, and YAGO do not inherently resolve question-specific ambiguity (Speer et al., 2017; Vrandecic and Krotzsch, 2014; Mahdisoltani et al., 2015; Garderes et al., 2020; Ravi et al., 2023).
- *Lack of structured contextual reasoning*: although LLM-based methods enable flexible reasoning through few-shot prompting (Yang et al., 2022; Tsimpoukelli et al., 2021), their predictions remain highly sensitive to input context. Without structured multimodal support, these models are prone to hallucination and unstable outputs (Wang et al., 2023).

To address these challenges, we propose a multi-level knowledge augmentation framework for KB-VQA based on a small-to-large model pipeline. Rather than introducing a new training algorithm or modifying the architecture of the LLM, our goal is to investigate *how multimodal evidence can be effectively constructed and organized at inference time* to support more reliable reasoning with a frozen LLM. In this work, “small-to-large evidence-guided collaboration” refers to an inference-time, context-level collaboration rather than parameter-level interaction or gradient-based knowledge transfer. Specifically, task-specific vision–language models act as upstream evidence constructors: they transform the input image-question pair into structured textual evidence, including question-oriented visual descriptions, entity-level clarifications, candidate answers, and contextual exemplars. These evidence blocks are then systematically organized and provided to the frozen LLM, which serves as the final reasoner. By coordinating perception, entity understanding, answer-space refinement, and contextual reasoning support within a unified evidence-construction process, the proposed framework improves the relevance and organization of multimodal evidence, thereby enhancing the reliability of knowledge-based reasoning.

Within this clarified scope, the contributions of this work are summarized as follows:

- We present a structured inference-time evidence augmentation framework for frozen-LLM-based KB-VQA. Rather than proposing a new backbone model or a new training algorithm, the framework focuses on organizing heterogeneous evidence sources into a coherent reasoning context for the final LLM.
- We decompose evidence construction into four complementary levels: question-oriented visual evidence, entity-level semantic clarification, candidate answer guidance, and contextual exemplar support. This decomposition specifies the role and organization of each evidence type, thereby making the prompt construction process more systematic than ad-hoc information concatenation.
- We provide an empirical study on OK-VQA and A-OKVQA, including module-wise ablations, statistical reliability analysis, and inference-cost accounting. We also discuss the limitations of the current evaluation and identify hallucination assessment, output-format reliability, and robustness under prompt perturbations as important directions for future work.

## 2 Methodology

Building upon the limitations identified in prior studies, we design a novel *multi-module inference-time evidence augmentation framework* that explicitly addresses the challenges of weak visual-semantic alignment, entity ambiguity, and insufficient contextual support. This section presents the overall architecture of our framework and details the role of each module.

### 2.1 Overall framework

**Figure 1:**
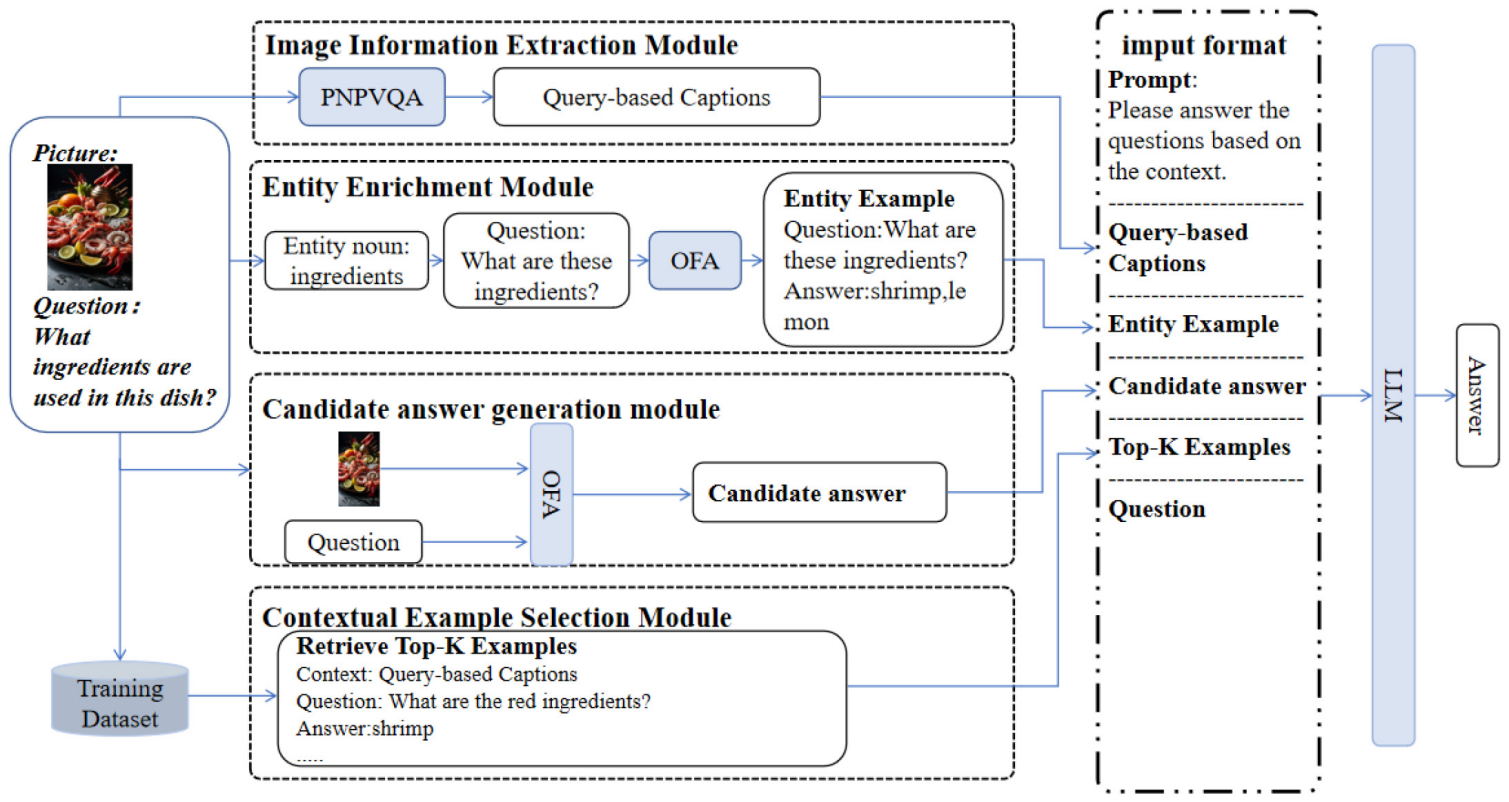
Overview of the proposed multi-module inference-time evidence augmentation framework.

As illustrated in Figure 1, the proposed framework adopts an inference-time small-to-large evidence-guided collaboration strategy. Here, “collaboration” does not mean that the smaller modules directly update or alter the parameters of the LLM. Instead, the smaller vision-language modules are responsible for constructing structured evidence from the input image-question pair, while the frozen LLM is responsible for final evidence-grounded reasoning. Specifically, the framework consists of four complementary components: (1) Image Information Extraction for generating question-oriented visual descriptions; (2) Entity Enhancement for producing entity-level clarifying cues; (3) Candidate Answer Generation for providing plausible answer references; and 4) Contextual Exemplar Retrieval for supplying relevant demonstrations. The outputs of these modules are organized into a unified textual prompt and provided to the LLM. This design enables the final reasoner to receive more relevant, less ambiguous, and better structured evidence without requiring parameter fine-tuning.

This architecture forms a structured perception–reasoning pipeline that integrates low-level visual cues with high-level semantic reasoning in a hierarchical manner. Given an input image and a natural language question, the answer is generated through four complementary steps.

- *The image information extraction module* produces question-oriented image descriptions and object labels.
- *The entity enhancement module* extracts key entities and formulates clarifying questions to obtain fine-grained semantic interpretations.
- *The candidate answer generation module* leverages pretrained vision-language models to supply diverse candidate answers as references.
- *The contextual exemplar retrieval module* selects the top-*K* most relevant exemplars from the training set and feeds them, along with the preceding information, into a large language model for reasoning.

Unlike conventional VQA pipelines, our framework does not merely fuse vision and language features. Instead, it introduces a multi-level context augmentation mechanism, where visual semantics, entity exemplars, candidate responses, and contextual demonstrations jointly guide LLM-based reasoning. This design enables the model to generate answers that are not only accurate but also semantically robust and contextually grounded.

It is important to distinguish the proposed framework from simple prompt concatenation. In our setting, the final input to the frozen LLM is indeed represented as a textual prompt, because the final reasoner is a text-based LLM. However, the key issue studied in this work is not merely appending more text to the prompt, but how to construct task-relevant evidence with explicit functional roles. Specifically, the four modules correspond to four different sources of uncertainty in KB-VQA: insufficient visual grounding, ambiguous entity semantics, unconstrained answer generation, and lack of task-specific reasoning demonstrations. The final prompt is therefore organized as a structured evidence context rather than an unstructured collection of auxiliary text. This design allows us to analyze the contribution of each evidence type through module-wise ablations and to study the trade-off between reasoning accuracy and inference cost.

### 2.2 Image information extraction module

To strengthen visual–semantic alignment, this module employs pretrained captioning and VQA-oriented models [e.g., PNP-VQA (Tiong et al., 2022)] to generate question-guided captions and detect key object labels. Unlike conventional image captioning, which produces generic descriptions, our design explicitly incorporates the question content, thereby generating targeted semantic cues. For example, for the query “What ingredients are used in this dish?”, the model may output “a dish containing shrimp, lemon, and vegetables,” which narrows the reasoning scope and improves subsequent inference (Hu et al., 2023). Formally, the extracted image information is represented as:


$$\begin{array}{l} F_{\text{img}}=f\left(I,Q\right)=\left\{D_{\text{img}},O\right\}. \end{array}$$ (1)

More specifically, *D*_img_ is represented as a set of question-oriented textual descriptions:


$$D_{\text{img}}=d_{\text{img}}^{1},d_{\text{img}}^{2},\ldots ,d_{\text{img}}^{K},$$

where each $d_{\text{img}}^{k}$ denotes a natural-language description generated under the guidance of the input question. Therefore, *D*_img_ is not a latent visual feature or an internal embedding, but a textual evidence block that can be directly inserted into the final LLM prompt. The object set *O* = {*o*_1_, *o*_2_, …, *o*_*n*_} consists of textual object labels detected or inferred from the image.

For the example shown in Table 1, *D*_img_ corresponds to the “Query-based Captions” field. Typical elements include “A table covered with sushi rolls and pastries” and “Sushi rolls are placed on top of cakes of different flavors and types.” Meanwhile, *O* may contain object-level concepts such as sushi rolls, pastries, cakes, candles, table, and vegetables. These textual descriptions and object labels jointly serve as question-relevant visual evidence for subsequent entity enhancement, candidate answer generation, and final LLM-based reasoning.

**Table 1:** An example for answer reasoning.

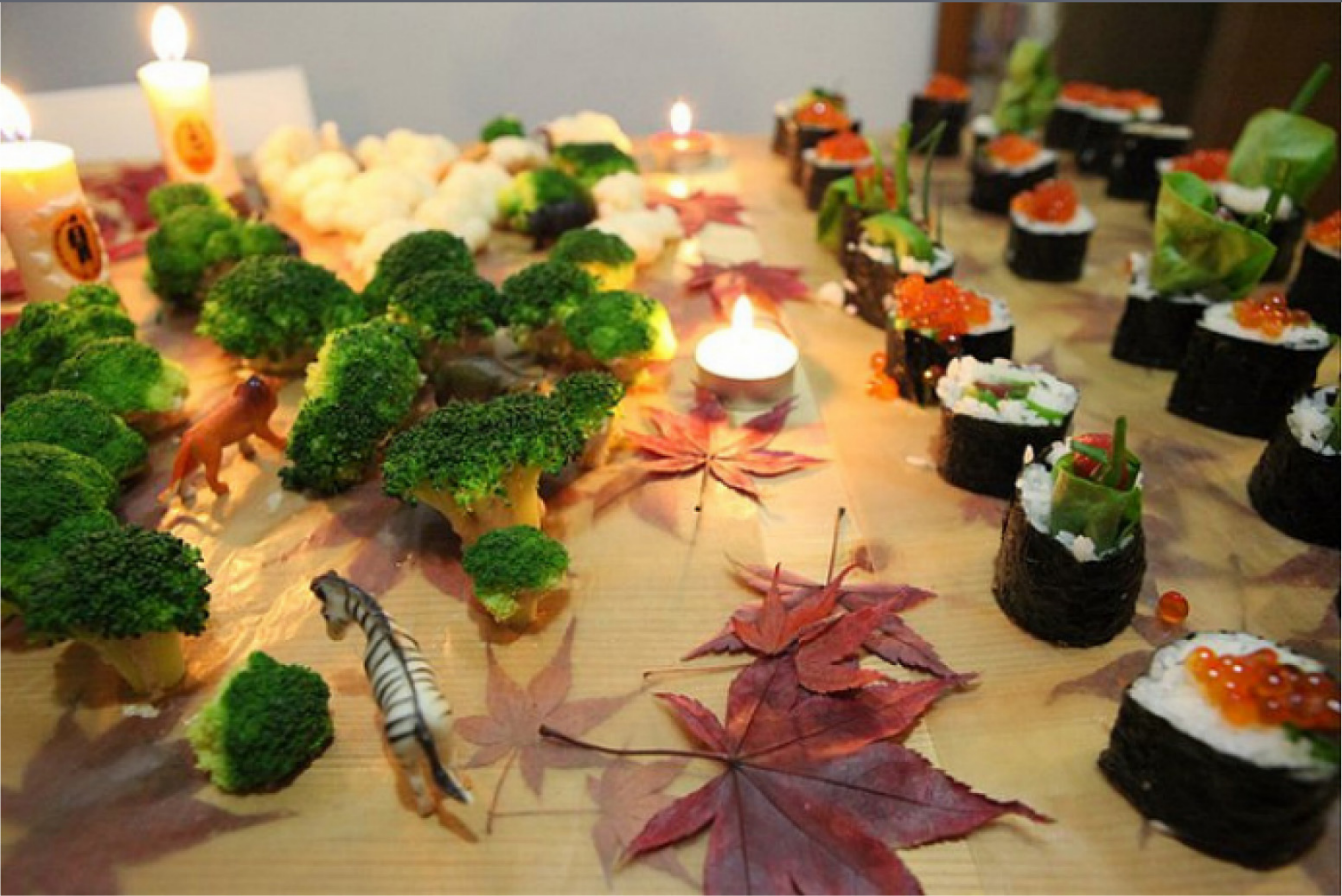	
Element	Content
User	What are the rolled black food items called?
Examples	The dishes are placed beside the vegetables in front of a table. A plate of appetizers wrapped with meat. Mini appetizers are placed on a small piece of food. The bamboo plates on the counter are filled with small portions of appetizers. These appetizers are ready to be eaten. *Image ID: 162760 Question: Do these snacks need to be cooked or can they be prepared raw? Answer: raw* (Other examples omitted for brevity.)
Query-based captions	A table covered with sushi rolls and pastries. The table is decorated with patterns of sushi rolls and various types of cakes. Sushi rolls are placed on top of cakes of different flavors and types. Cakes with candles have sushi rolls and rolled combinations placed on them. Sushi rolls can be arranged in rolls as display items for sushi.
Entity hints:	What are the foods in the picture? -> sushi and cauliflower
Candidate answers	sushi mats sake rolls roll

As shown in Table 2, the “Query-based Captions” correspond to *D*_img_, the “Entity Hints” correspond to *E*_ent_, and the “Candidate Answers” correspond to *A*_cand_.

**Table 2:** Notation and concrete representations of intermediate module outputs.

Symbol	Source module	Concrete representation	Example
*D*_img_	Image information extraction	A set of question-oriented natural-language image descriptions	“A table covered with sushi rolls and pastries.”
*O*	Image information extraction	A set of textual object labels detected or inferred from the image	sushi roll, cake, candle, table
*E*	Entity extraction	Key entities extracted from the input question	rolled black food items
*Q*_ent_	Entity enhancement	Entity-oriented clarifying sub-question	“What are the foods in the picture?”
*E*_ent_	Entity enhancement	Textual auxiliary cue generated for the clarifying question	“sushi and cauliflower”
*A*_cand_	Candidate answer generation	A list of plausible candidate answers used as soft guidance	sushi, sake rolls, roll
*E*_ctx_	Contextual exemplar retrieval	Retrieved textual triplets consisting of image description, question, and answer	Ĩ_*i*_, *Q*_*i*_, *A*_*i*_
*P*″	Final inference	Instruction prompt used to organize all evidence blocks for LLM reasoning	“Answer the question based on the following context.”

Bold values indicate the highest accuracy achieved among the compared methods.

### 2.3 Entity enhancement module

While question-guided captions provide useful global context, they may still miss fine-grained semantic entities. To alleviate this issue, we first extract salient entities from the input question, denoted as


$$\begin{array}{l} E=\left\{e_{1},e_{2},\ldots ,e_{m}\right\}. \end{array}$$ (2)

Based on these entities, we construct clarifying sub-questions and use OFA (Wang et al., 2022) to obtain image-grounded auxiliary cues:


$$\begin{array}{l} E_{\text{ent}}=\text{OFA}\left(I,Q_{\text{ent}}\right), \end{array}$$ (3)

where *Q*_ent_ denotes the entity-oriented clarifying question and *E*_ent_ denotes the resulting entity cues. In this work, the entity enhancement module is used as an auxiliary source of evidence rather than a guaranteed source of truth. In some cases, automatically generated clarifying questions may be imperfect or partially hallucinated. We therefore interpret this module as a mechanism that can reduce entity ambiguity in many cases, rather than one that always resolves it completely. A quantitative analysis of invalid or misleading clarifications is left for future work.

### 2.4 Candidate answer generation module

Even with enhanced descriptions and entity cues, answer generation may remain under-constrained. To provide additional guidance, we use OFA (Wang et al., 2022) to produce a set of plausible candidate answers:


$$\begin{array}{l} A_{\text{cand}}=\text{OFA}\left(I,Q\right)=\left\{a_{1},a_{2},\ldots ,a_{r}\right\}. \end{array}$$ (4)

These candidates are supplied to the frozen LLM as reference information. They are not treated as hard constraints; instead, they function as soft guidance that helps narrow the possible answer space and reduce irrelevant generations. We further note that candidate-answer guidance may introduce bias toward outputs preferred by the candidate generator. For this reason, the final answer is still generated by jointly considering candidate answers together with question-oriented image evidence, entity cues, and retrieved contextual exemplars.

### 2.5 Contextual exemplar retrieval module

To improve in-context reasoning, we retrieve relevant training exemplars for each input sample and provide them to the frozen LLM as contextual demonstrations.

(1) *Training-only retrieval bank*: the retrieval bank is constructed using only the training split. Validation and test instances are never inserted into the retrieval bank. This design avoids direct data leakage from evaluation samples into the prompt context.

(2) *Feature extraction*: given an input sample (*I, Q*) and a candidate training sample (*I*′, *Q*′), we extract visual and textual features using BLIP (Li et al., 2022):


$$\begin{array}{l} v=\text{BLI}{\text{P}}_{v}\left(I\right),\;t=\text{BLI}{\text{P}}_{q}\left(Q\right), \end{array}$$ (5)

$$\begin{array}{l} v^{\prime }=\text{BLI}{\text{P}}_{v}\left(I^{\prime }\right),\;t^{\prime }=\text{BLI}{\text{P}}_{q}\left(Q^{\prime }\right). \end{array}$$ (6)

Here, BLIP*v*(·) and BLIP*q*(·) denote the visual and textual encoders of BLIP, respectively. Both the visual feature *v* and the textual feature *t* are taken from the projected multimodal embedding space of BLIP. In our implementation, the projection dimension is 256, i.e., *v, t, v*′, *t*′∈ℝ^256^. Before computing the retrieval score, all feature vectors are *L*_2_-normalized:


$$\bar{v}=\frac{v}{|v{|}_{2}},\;\bar{t}=\frac{t}{|t{|}_{2}},\;{\bar{v}}^{\prime }=\frac{v^{\prime }}{|v^{\prime }{|}_{2}},\;{\bar{t}}^{\prime }=\frac{t^{\prime }}{|t^{\prime }{|}_{2}}.$$

(3) *Similarity computation*: the similarity score in Equation 7 is therefore computed based on normalized embeddings, where the cosine similarity is equivalent to the inner product between unit vectors. This normalization also ensures that the visual and textual similarities are on the same scale before they are averaged.


$$\begin{array}{l} s\left(\left(I,Q\right),\left(I^{\prime },Q^{\prime }\right)\right)=\frac{1}{2}\left[{\bar{v}}^{⊤}{\bar{v}}^{\prime }+{\bar{t}}^{⊤}{\bar{t}}^{\prime }\right], \end{array}$$ (7)

where $\bar{v}$, ${\bar{v}}^{\prime }$, $\bar{t}$, and ${\bar{t}}^{\prime }$ denote the *L*_2_-normalized visual and textual embeddings. Since all embeddings are normalized to unit length, ${\bar{v}}^{⊤}{\bar{v}}^{\prime }$ and ${\bar{t}}^{⊤}{\bar{t}}^{\prime }$ are equivalent to the cosine similarities cos(*v, v*′) and cos(*t, t*′), respectively. The final retrieval score is the average of image–image similarity and question–question similarity.

(4) *Exemplar selection and formatting*: based on the retrieval score, we rank all candidate training samples and select the top-*K* exemplars. Each exemplar is converted into a textual triplet


$$\begin{array}{l} E_{\text{ctx}}={\left\{\left({Ĩ}_{i},Q_{i},A_{i}\right)\right\}}_{i=1}^{K}, \end{array}$$ (8)

where Ĩ_*i*_ denotes a concise textual description of the exemplar image, *Q*_*i*_ is the associated question, and *A*_*i*_ is the corresponding answer. This textualization step is adopted because the final reasoner is a frozen text LLM. We acknowledge that converting exemplar images into text descriptions discards raw visual modality during in-context learning; therefore, this design should be viewed as a practical interface choice rather than a fully multimodal prompting solution.

### 2.6 Fusion and final inference

After obtaining the outputs from all preceding modules—including image descriptions, entity exemplars, candidate answers, and contextual exemplars—we concatenate these signals with the task instruction to construct a unified input prompt for the LLaMA2 language model (Touvron et al., 2023). Similar to recent training-free or lightly coupled multimodal reasoning paradigms (Yang et al., 2022; Tiong et al., 2022; Xenos et al., 2023), our framework keeps the LLM parameters frozen, and improvements are achieved through structured context design rather than parameter fine-tuning. At decoding step *t*, the prediction of the LLM is defined as:


$$\begin{array}{l} {ŷ}_{t}=\arg\underset{y_{t}}{\max}P_{\text{LLM}}\left(y_{t}|{ŷ}_{<t},P^{\prime \prime },F_{\text{img}},E_{\text{ent}},{\text{Ans}}_{\text{candidate}},E_{\text{ctx}}\right), \end{array}$$ (9)

where ŷ_*t*_ is the token generated at step *t*, ŷ_<*t*_ represents the sequence of previously generated tokens, and *P*″ is the instruction prompt (e.g., “Answer the question based on the following context”). Here, *F*_img_ corresponds to the image descriptions and object labels, *E*_ent_ denotes the entity exemplars, Ans_candidate_ represents the candidate answer set, and *E*_ctx_ is the set of contextual exemplars. The final answer is generated autoregressively until an end-of-sequence token is produced.

This fusion stage reflects the central design of our framework: by unifying multi-level cues—visual semantics, entity clarifications, candidate references, and exemplar demonstrations—the LLM is guided to generate responses that are not only accurate, but also semantically coherent, robust against ambiguity, and well-grounded in contextual knowledge. An example of answering the question is shown in Table 1. As shown in this table, given an input image and question, the model first generates question-oriented captions and extracts key entities. It then produces clarifying cues and candidate answers using vision-language models. Finally, relevant contextual exemplars are retrieved and combined with all intermediate signals to guide the LLM for answer generation. This example highlights how multi-level information is progressively integrated for structured reasoning. To improve output stability, we use a multi-query ensemble strategy during inference. Specifically, each test sample is queried three times with closely related prompt variants, and the final prediction is obtained through consistency-based aggregation. The three prompt variants share the same structured evidence, including *F*_img_, *E*_ent_, *A*_cand_, and *E*_ctx_, and differ only in the wording of the instruction prompt *P*″:

- *Variant 1*: answer the question based on the following context. Give only a short answer.
- *Variant 2*: using the image descriptions, entity hints, candidate answers, and the examples above, answer the question with a concise phrase.
- *Variant 3*: read the provided context carefully and output the most likely short answer to the question.

These variants are intentionally minor paraphrases: they keep the evidence and task fixed while perturbing only the surface form of the instruction. The final answer is obtained by majority voting over the three generated outputs; if all three outputs are different, we select the first generated answer after answer normalization. This strategy is intended as an inference-time enhancement rather than a training procedure. It improves output stability but also increases computational cost and latency, which we analyze in Section 3.6.

Although the multi-query ensemble improves output stability, it also increases inference latency because each test sample is queried multiple times. In addition, the structured evidence blocks make the final prompt longer than a standard LLM prompt. Since the LLM remains frozen, the proposed method does not introduce additional training or parameter-update cost, but it does increase inference-time computation. Thus, our framework should be regarded as an accuracy-oriented evidence augmentation strategy rather than a deployment-optimized solution. The efficiency can be improved by disabling ensemble inference, reducing the number of retrieved exemplars, or shortening the generated evidence.

### 2.7 Discussion

To mitigate the potential risk of introducing incorrect candidates or inherent model biases into the LLM input, we leverage the In-Context Learning capability of the LLM. By providing diverse contextual exemplars and instruction-based prompts, the LLM is encouraged to cross-reference the visual descriptions with the generated candidates. While the current framework relies on the LLM's internal reasoning to filter out noise, we acknowledge that incorporating an explicit confidence scoring mechanism or a verification module for candidate answers could further enhance the reliability of the reasoning process, which we leave for future exploration.

## 3 Evaluation and analysis

To rigorously validate the effectiveness of the proposed framework, we conduct a comprehensive set of experiments, including overall performance evaluation, ablation studies, and qualitative case analyses.

### 3.1 Datasets

We evaluate the proposed model on two widely used knowledge-based VQA benchmarks: OK-VQA and A-OKVQA, both of which require reasoning beyond visual recognition by incorporating external knowledge. (1) OK-VQA: This dataset is one of the most popular benchmarks for knowledge-intensive VQA research (Marino et al., 2019). The questions are open-domain and explicitly designed to require external knowledge for correct answering. All images are drawn from the MSCOCO dataset (Lin et al., 2014), containing approximately 80, 000 images for training and 40, 000 for testing. In total, OK-VQA provides 14, 055 questions, with 9, 009 questions for training and 5, 046 for testing. Each question is annotated with five human-provided answers, ensuring a diverse ground-truth reference. (2) A-OKVQA: As the largest publicly available KB-VQA dataset to date (Schwenk et al., 2022), A-OKVQA provides a more challenging benchmark. It is split into training (17*k*), validation (1.4*k*), and testing (6.7*k*) subsets. The dataset defines two tasks:

- DA (Direct Answer): an open-ended setting requiring free-form answers (similar to OK-VQA).
- MC (Multiple Choice): a classification setting where the correct answer must be selected from four candidates.

In this study, we focus exclusively on the DA task, as it is closer to real-world applications of knowledge-based reasoning.

### 3.2 Evaluation metric

All results are evaluated using the standard VQA accuracy metric, where a prediction is considered correct if it matches at least three out of five human-provided answers. This metric accounts for the inherent variability of open-ended responses and enables fair comparison with prior methods. Specifically, we use the official accuracy metric (Antol et al., 2015), defined as:


$$\begin{array}{l} \text{Accuracy}\left(\text{ans}\right)=\min\left(\frac{\#\text{ans}}{3},1\right), \end{array}$$ (10)

where ans denotes the predicted answer and #ans denotes the number of annotators whose answers match the prediction.

While VQA accuracy is the primary metric in this study, a single accuracy value does not indicate the reliability of a comparison on a finite test set. We therefore complement accuracy with two statistical analyses that can be derived from the reported scores and the known evaluation-set sizes. First, we report the 95% Wilson confidence interval for the overall accuracy, using *N* = 5, 046 for OK-VQA and (*N*≈6, 700) for the A-OKVQA direct-answer split. This interval quantifies the estimation uncertainty of each reported accuracy. Second, for comparisons between the Full model and ablated variants, we conduct an approximate two-proportion *z*-test and report the corresponding *z* statistic and *p*-value. Since the standard VQA score is a soft metric, $\min\left(\frac{\#\text{ans}}{3},1\right)$, rather than a strict binary outcome, these statistical tests should be interpreted as indicative rather than exact.

We also acknowledge that accuracy does not fully capture all reliability aspects of frozen-LLM-based KB-VQA systems. Hallucination, output-format errors, inference latency, and robustness to prompt variations are also important evaluation dimensions. In the current revision, we explicitly analyze inference cost in Section 3.6. However, rigorous quantitative evaluation of hallucination and output-format errors would require additional manual annotation or carefully designed task-specific criteria for open-ended answers. Therefore, we discuss these limitations qualitatively and leave systematic evaluation of hallucination, format reliability, and prompt robustness to future work.

### 3.3 Comparison methods

To demonstrate the effectiveness of the proposed model, we compare it against a broad range of representative baselines, covering heuristic prompting, pretrained multimodal models, and in-context learning strategies:

- *PICa-Full* (Yang et al., 2022): a GPT-3-based KB-VQA method that prompts the language model with image captions and in-context examples.
- *Prophet* (Shao et al., 2023): a prompting-based KB-VQA model that provides LLMs with answer-oriented heuristics to improve prediction accuracy and robustness.
- *VIGC* (Wang et al., 2024): a self-instruct multimodal framework in which the model generates and corrects visual instructions to improve downstream performance.
- *SmOLa (Omni-SMoLA)* (Wu et al., 2024): a compact multimodal model that improves generalist reasoning through soft mixtures of low-rank experts.
- *InstructBLIP (Vicuna-7B)* (Dai et al., 2023): a multimodal architecture that combines large-scale vision–language pretraining with instruction tuning for stronger generalization.
- *PaLI* (Chen et al., 2023): a large-scale unified multimodal model capable of handling diverse vision–language tasks including VQA.
- *VPD (visual program distillation)* (Hu et al., 2024): a framework that distills tool use and programmatic reasoning into vision–language models.
- *FIIG (Ensemble)* (Wang et al., 2023): a filtering-augmented VQA approach in which LLMs proactively raise clarifying questions about images and combine them with filtering strategies.
- *Simple baseline* (Xenos et al., 2023): a strong in-context KB-VQA baseline built on LLaMA with question-informative captions.

These baselines collectively represent strong and recent directions in knowledge-based VQA, against which we benchmark the proposed model under identical experimental settings.

### 3.4 Experimental environment

All experiments are implemented in PyTorch and executed on a workstation equipped with 2 × NVIDIA RTX 4090 GPUs (24 GB each). We adopt LLaMA2 (13B) (Touvron et al., 2023) as the frozen backbone LLM, ensuring that performance improvements stem from our framework rather than parameter fine-tuning. For each test instance, we retrieve 10 contextual exemplars and generate nine question-guided captions. To further enhance stability, we adopt a multi-query ensemble strategy: each input is prompted three times using the three instruction variants described in Section 2.6, and the final prediction is obtained by consistency-based majority voting over the three outputs. This strategy reduces randomness and improves the robustness of inference. We report the resulting computational overhead explicitly in Section 3.6.

### 3.5 Experimental results and analysis

#### 3.5.1 Comparison with baseline methods on the OK-VQA dataset

The results on OK-VQA (Table 3) reveal several notable trends. First, GPT-3–based generative approaches, including PICa-Full, Prophet, and FIIG (Ensemble) (Yang et al., 2022; Shao et al., 2023; Wang et al., 2023), achieve accuracies ranging from 48.0% to 61.3%. Although Prophet (61.1%) and FIIG (61.3%) demonstrate improvements through heuristic prompting and ensemble strategies, their reliance on language-only prompting limits their ability to capture fine-grained visual semantics.

**Table 3:** Comparison with existing methods on knowledge resources and accuracy.

Methods	Knowledge resources	Accuracy
PICa-Full	GPT-3	48.00
Prophet	GPT-3	61.10
FIIG (Ensemble)	GPT-3	61.30
SmOLa	PALI-X	62.40
VIGC	InstructBLIP	63.80
PaLI	PaLI	64.50
VPD	PALI-X	64.60
Ours (Base)	LLaMA2	62.57
**Ours (Full)**	LLaMA2	**66.72**

Bold values indicate the highest accuracy achieved among the compared methods.

In contrast, multimodal pretrained or instruction-tuned models such as SmOLa, VIGC, PaLI, and VPD (Wu et al., 2024; Wang et al., 2024; Chen et al., 2023; Hu et al., 2024) achieve stronger results. These improvements highlight the effectiveness of multimodal pretraining and instruction tuning in bridging vision and language.

Our proposed model achieves 62.57% in the Base setting, already outperforming most GPT-3-based models and approaching the performance of PaLI. More importantly, by introducing entity enhancement and candidate answer generation, the Full model achieves 66.72%, surpassing the strongest baseline (VPD, 64.6%) by 2.12 percentage points. This performance gain demonstrates that the modular design effectively reduces entity ambiguity and enriches contextual reasoning, leading to more precise and knowledge-grounded answers.

#### 3.5.2 Comparison with baseline methods on the A-OKVQA dataset

**Figure 2:**
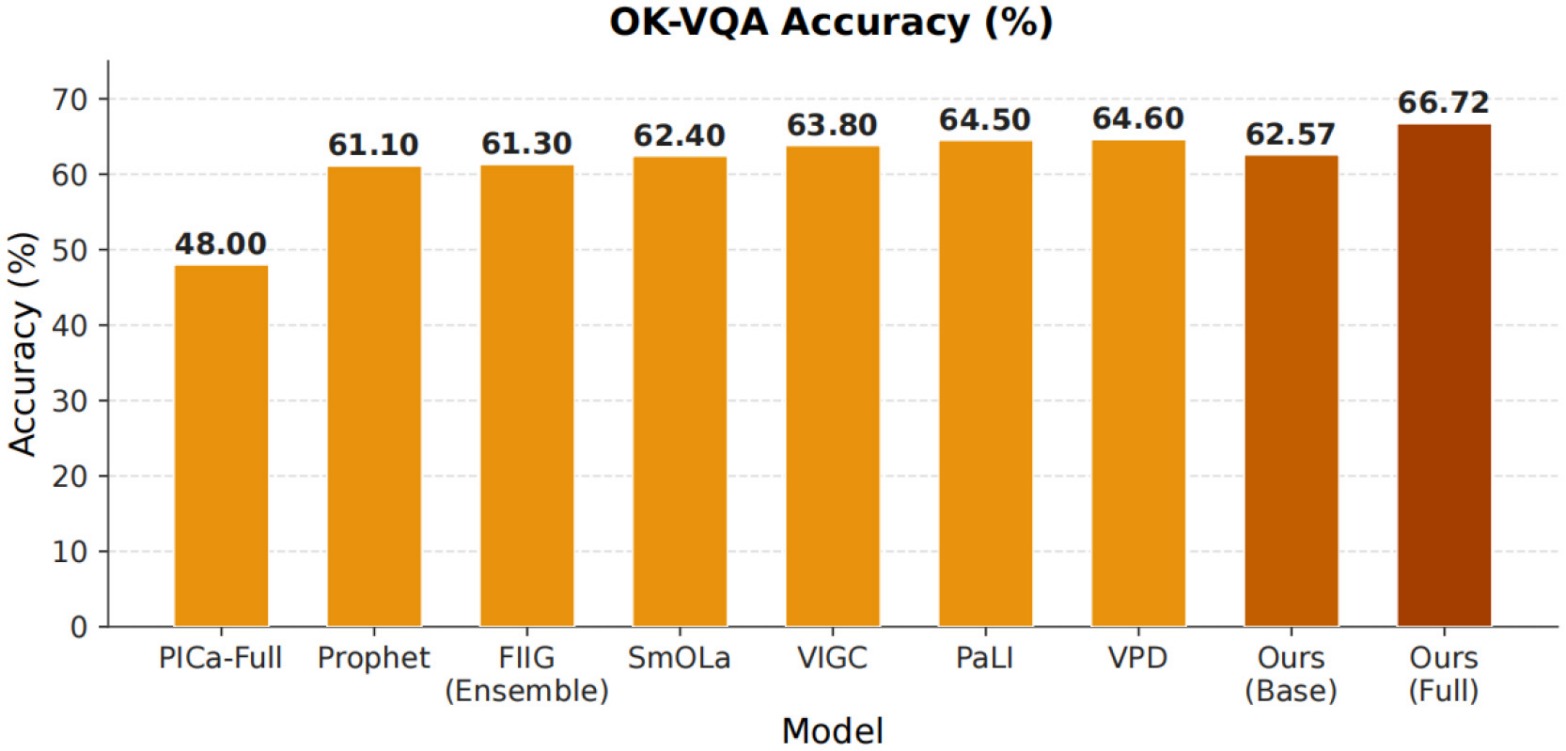
Bar chart illustrating the experimental results on the OK-VQA dataset.

The A-OKVQA dataset provides a more challenging benchmark due to its larger scale and broader coverage of knowledge-intensive questions. The results are illustrated in Figure 2. As shown in Table 4, GPT-3-based models again face clear bottlenecks: Prophet reaches 58.2% and FIIG (Ensemble) achieves 59.8%, indicating only marginal improvements over their OK-VQA performance. This trend suggests that heuristic prompting alone struggles to generalize effectively across datasets with increased reasoning complexity.

**Table 4:** Comparison with existing methods on knowledge resources and accuracy.

Methods	Knowledge resources	Accuracy
LLaMA2	LLaMA2	57.10
Prophet	GPT-3	58.20
FIIG (Ensemble)	GPT-3	59.80
InstructBLIP (Vicuna-7B)	InstructBLIP	64.00
VIGC	InstructBLIP	64.10
VPD	PALI-X	62.70
SmOLa	PALI-X	65.30
Ours (Base)	LLaMA2	66.32
**Ours (Full)**	LLaMA2	**69.51**

**Figure 3:**
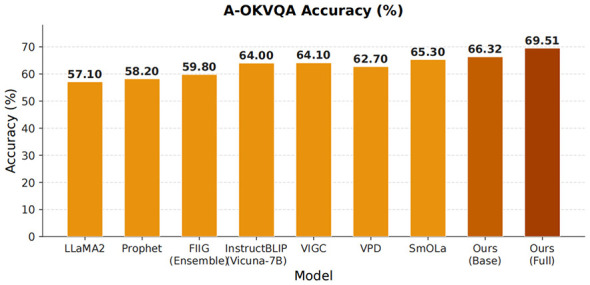
Bar chart illustrating the experimental results on the A-OKVQA dataset.

Multimodal pretrained models deliver more competitive results. The results are illustrated in Figure 3. InstructBLIP, VIGC, VPD, and SmOLa (Dai et al., 2023; Wang et al., 2024; Hu et al., 2024; Wu et al., 2024) all outperform the GPT-3-only prompting baselines, further confirming the advantages of multimodal instruction tuning and integrated reasoning mechanisms.

Our proposed model demonstrates superior performance in both configurations. The Base model achieves 66.32%, already outperforming all baselines, including SmOLa. When enhanced with entity exemplars and candidate answers, the Full model further improves to 69.51%, exceeding the strongest baseline by 4.21 percentage points. This consistent improvement across datasets underscores two key strengths of our approach: (a) the ability of entity enhancement to resolve semantic ambiguity in complex questions, and (b) the effectiveness of candidate answer guidance in stabilizing reasoning and reducing error propagation. These findings validate the robustness and generalization capacity of the proposed multi-module framework in diverse, knowledge-intensive VQA settings.

### 3.6 Ablation studies

**Figure 4:**
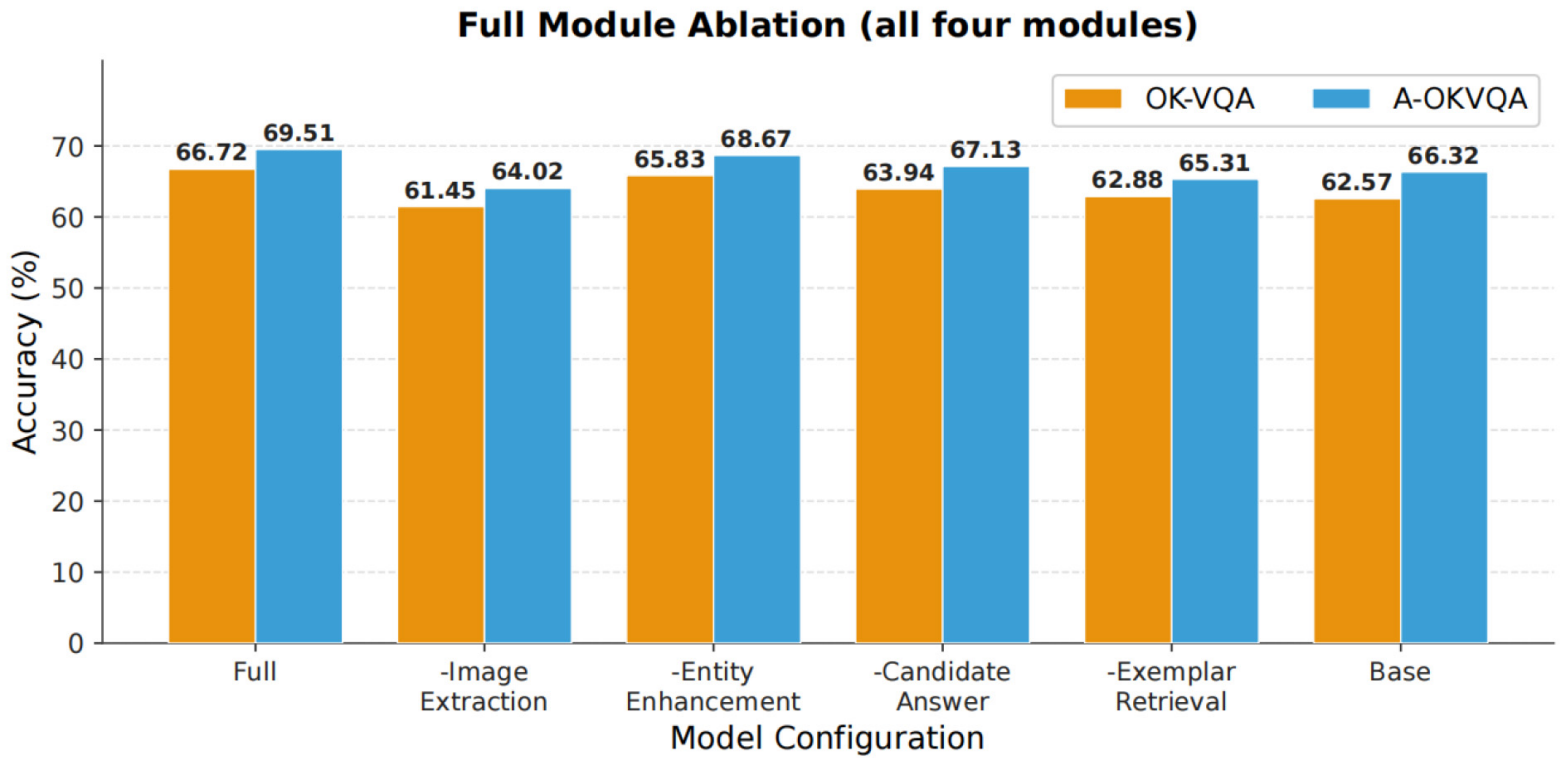
Ablation results of different model variants on OK-VQA and A-OKVQA. The legend and numerical annotations are arranged to avoid overlap and improve readability.

To examine the contribution of each component, we conduct ablation experiments on OK-VQA and A-OKVQA. In addition to the originally analyzed *Entity Enhancement* and *Candidate Answer* modules, we further evaluate the effects of *Image Information Extraction* and *Contextual Exemplar Retrieval*. Specifically, -Image Extraction” replaces question-oriented captions with a single generic caption, while -Exemplar Retrieval” removes the retrieved in-context demonstrations. The complete results are reported in Table 5 and visualized in Figure 4.

**Table 5:** Impact of different modules on OK-VQA and A-OKVQA.

Model	OK-VQA (*N* = 5046)		A-OKVQA (*N*≈6700)		Significant?
	Acc. / Δ	*z* / *p*	Acc. / Δ	*z* / *p*	
Full	66.72%/–	– / –	69.51%/–	– / –	–
-Image extraction	61.45%/5.27	5.53/ <0.001	64.02%/5.49	6.76/ <0.001	Yes
-Entity enhancement	65.83%/0.89	0.95/0.344	68.67%/0.84	1.05/0.293	No
-Candidate answer	63.94%/2.78	2.94/0.003	67.13%/2.38	2.96/0.003	Yes
-Exemplar retrieval	62.88%/3.84	4.04/ <0.001	65.31%/4.20	5.19/ <0.001	Yes
Base	62.57%/4.15	4.36/ <0.001	66.32%/3.19	3.96/ <0.001	Yes

“Full” is the complete model; each “-*X*” row removes one module; “Base” removes both Entity Enhancement and Candidate Answer. Δ is the absolute accuracy drop relative to Full. The *z* statistic and *p*-value are from a two-proportion test against Full on the corresponding fixed test set.

To address the concern that small accuracy differences may be caused by randomness, we further provide an approximate statistical analysis based on the existing ablation results. This analysis does not require additional model runs or new annotations. Since both benchmarks have fixed evaluation sets, we use an approximate two-proportion *z*-test to assess whether the observed differences between the Full model and each ablated variant are statistically reliable. Because the standard VQA accuracy is a soft score rather than a strict binary outcome, the resulting *p*-values should be interpreted as indicative rather than exact.

The results show that Image Information Extraction is the most influential component. Removing this module causes the largest performance degradation, indicating that question-oriented visual descriptions serve as the key visual-to-text bridge for the frozen LLM. Without such question-relevant visual evidence, the LLM has difficulty grounding its answer in the image content.

Contextual Exemplar Retrieval and Candidate Answer Generation also contribute substantially. Removing the exemplar retrieval module degrades performance, suggesting that retrieved demonstrations provide useful task-specific answer formats and reasoning patterns for in-context learning. Removing the candidate answer module also leads to a clear drop, showing that candidate answers act as soft constraints that help narrow the answer space and reduce irrelevant generations.

By contrast, the effect of Entity Enhancement is relatively modest. Removing this module decreases accuracy by only 0.89 percentage points on OK-VQA and 0.84 percentage points on A-OKVQA. The approximate statistical analysis suggests that these differences are not statistically significant. Therefore, we do not claim that Entity Enhancement is a decisive component of the framework. Instead, we interpret it as a secondary complementary module that may provide useful entity-level cues in some ambiguous cases, while its overall contribution is weaker than that of the other three modules.

Overall, the ablation study provides a more balanced understanding of the proposed framework. Image Information Extraction, Contextual Exemplar Retrieval, and Candidate Answer Generation are the main contributors to the overall improvement, while Entity Enhancement offers a modest auxiliary refinement.

We summarize four findings.

(1) Removing the image information extraction module causes the largest degradation on both datasets (5.27 and 5.49 points, both *p* < 0.001), confirming that question-oriented visual evidence is the foundational “visual-to-text” bridge: without it, the frozen LLM lacks the fine-grained, question-specific cues required for grounded reasoning.

(2) Removing the contextual exemplar retrieval module also leads to a large and significant drop (3.84 and 4.20 points, both *p* < 0.001), indicating that retrieved demonstrations are essential for supplying the task-specific format and reasoning pattern needed for in-context learning.

(3) Removing the candidate answer module yields a significant degradation (2.78 and 2.38 points, *p*≈0.003) that is larger than that of Entity Enhancement, showing that constraining the answer space has a stronger direct influence on final predictions.

(4) The drop from removing entity enhancement is small (0.89/0.84 points) and, under the two-proportion test, is *not* statistically significant (*p*≈0.34 on OK-VQA and *p*≈0.29 on A-OKVQA). We therefore describe Entity Enhancement honestly as a helpful but secondary, complementary refinement rather than a decisive module, while the other three modules each provide statistically significant gains. In summary, the complete ablation confirms that all four modules contribute positively, with Image Extraction and Exemplar Retrieval being the most critical and Candidate Answer providing the strongest significant boost among the two originally ablated modules.

### 3.7 Inference cost analysis

Because our framework adds several auxiliary modules and a multi-query ensemble, a natural concern is whether it substantially increases computation. Instead of reporting hardware-dependent wall-clock latency, we provide a model-level inference-cost accounting based on the pipeline structure. This analysis clarifies which components introduce additional overhead and which costs can be amortized or removed in efficiency-sensitive settings.

Two conclusions follow without any additional measurement. First, the evidence-construction modules add only a fixed, bounded overhead per question: the small vision-language models (PNP-VQA, OFA, BLIP) are run a constant number of times and are each far smaller than the frozen LLaMA2-13B backbone, while the retrieval bank features are computed once offline and the per-query ranking is a set of vectorized dot products whose cost is negligible relative to a single LLM forward pass. Second, the dominant marginal cost is the LLM itself, and the only component that multiplies it is the multi-query ensemble, which raises the number of LLaMA2-13B decoding passes from 1 to 3 and thus roughly triples the LLM-side cost. We therefore characterize the framework explicitly as an inference-time augmentation strategy that trades extra computation for higher and statistically significant accuracy; when latency is the priority, the single-query Full configuration removes the 3 × decoding factor while retaining the structured-evidence design, leaving only the modest, fixed small-model overhead. A precise wall-clock and FLOPs profiling on specific hardware is left as part of the computational-cost study identified in the conclusion (Table 6).

**Table 6:** Per-question inference-cost accounting of the proposed framework.

Component	Model	Invocations per question
Image information extraction	PNP-VQA (small)	Nine caption generations + label detection
Entity enhancement	OFA (small)	One clarifying query
Candidate answer generation	OFA (small)	One candidate query
Exemplar retrieval – encoding	BLIP (small)	Two encoder passes (image + question)
Exemplar retrieval – bank features	BLIP (small)	Offline (precomputed for the training split)
Exemplar retrieval – ranking	–	*O*(*N*_train_) cosine scores (vectorized, negligible)
Fusion and inference	LLaMA2-13B (large)	Three decoding passes (single-query: 1)

### 3.8 Comparative analysis

Beyond the ablation perspective, it is worth situating our proposed framework against external baselines. On OK-VQA, the Base model (62.57%) already surpasses GPT-3-based approaches such as Prophet (61.1%) and FIIG (61.3%), reflecting the benefit of question-guided image extraction and exemplar retrieval. The Full model further improves to 66.72%, yielding a 4.15-point gain over Base and outperforming the strongest baseline, VPD (64.6%), by 2.12 points.

On A-OKVQA, the Base setting (66.32%) is competitive with or better than large multi-modal models like SmOLa (65.3%). The Full configuration reaches 69.51%, surpassing Base by 3.19 points and outperforming the best baseline by 4.21 points.

Taken together, these results reveal a consistent pattern: even without auxiliary modules, our framework is competitive with state-of-the-art methods, and the integration of entity enhancement and candidate answer generation provides further, significant gains. This confirms that the modular design not only improves raw accuracy but also equips the model with robustness and adaptability for complex, knowledge-intensive VQA tasks.

## 4 Conclusions

This paper presents a multi-level knowledge augmentation framework for knowledge-based visual question answering under a frozen-LLM reasoning paradigm. Rather than modifying the underlying LLM, the proposed method constructs structured evidence for the final reasoner by integrating question-oriented image information, entity-level auxiliary cues, candidate answer guidance, and contextual exemplars. The results demonstrate that organizing complementary evidence sources can improve KB-VQA performance by strengthening visual–semantic alignment, alleviating entity-level ambiguity, and providing more informative context for knowledge-grounded inference.

This work should be viewed as a modular inference-time evidence augmentation framework rather than a lightweight deployment-ready solution. Although the current evaluation focuses primarily on task accuracy, we acknowledge that hallucination, output-format reliability, inference latency, and robustness to prompt or retrieval perturbations are also important for frozen-LLM-based KB-VQA. Future work will therefore focus on improving computational efficiency, developing more systematic reliability-oriented evaluation metrics, enhancing prompt and retrieval robustness, and extending the framework to broader multimodal reasoning tasks.

## Data Availability

The original contributions presented in the study are included in the article/supplementary material, further inquiries can be directed to the corresponding author.
